# Decoding the Roles of Long Noncoding RNAs in Hepatocellular Carcinoma

**DOI:** 10.3390/ijms22063137

**Published:** 2021-03-19

**Authors:** Lok-Sze Wong, Chun-Ming Wong

**Affiliations:** The State Key Laboratory of Liver Research, Department of Pathology, Li-Ka Shing Faculty of Medicine, The University of Hong Kong, Hong Kong, China; lokszececi@gmail.com

**Keywords:** long non-coding RNA, hepatocellular carcinoma, cancer hallmarks, biomarkers, therapeutic strategies, HBV/HCV infections

## Abstract

Hepatocellular carcinoma (HCC) is one of the most prevalent malignancies worldwide. HCC is associated with several etiological factors, including HBV/HCV infections, cirrhosis, and fatty liver diseases. However, the molecular mechanism underlying HCC development remains largely elusive. The advent of high-throughput sequencing has unveiled an unprecedented discovery of a plethora of long noncoding RNAs (lncRNAs). Despite the lack of coding capacity, lncRNAs have key roles in gene regulation through interacting with various biomolecules. It is increasingly evident that the dysregulation of lncRNAs is inextricably linked to HCC cancer phenotypes, suggesting that lncRNAs are potential prognostic markers and therapeutic targets. In light of the emerging research in the study of the regulatory roles of lncRNAs in HCC, we discuss the association of lncRNAs with HCC. We link the biological processes influenced by lncRNAs to cancer hallmarks in HCC and describe the associated functional mechanisms. This review sheds light on future research directions, including the potential therapeutic applications of lncRNAs.

## 1. Introduction

Hepatocellular carcinoma (HCC) is one of the most prevalent cancers worldwide. It is particularly prevalent in Asia and Africa where 80% of HCC cases are associated with viral hepatitis [1]. Other risk factors for HCC include excessive alcohol consumption, dietary exposure to aflatoxin, non-alcoholic fatty liver disease (NAFLD), and liver cirrhosis [2]. Owing to diagnostic delay and limited effective treatment options, the mortality rate of HCC is high, with over 600,000 global deaths annually [3]. A minority of HCC patients (10–20%) who present with HCC at early stages can be treated with surgical resection and liver transplantation. Unfortunately, due to the asymptomatic nature of HCC, most HCC patients are diagnosed at advanced stages, during which surgical therapy is no longer an option. Treatments such as trans-arterial chemoembolization (TACE) and systemic chemotherapies are the current mainstay of therapy for advanced HCC patients [4]. To date, multi-tyrosine kinase inhibitors such as Sorafenib, Lenvatinib, and Regorafenib, have been approved for advanced HCC treatment, yet they can only extend survival for three months [5]. Given the poor survival and high recurrence rate of HCC, researchers are now endeavoring to study the underlying molecular landscape of HCC progression to develop new treatment strategies for HCC patients. 

The progression of HCC is a multistep process driven by the accumulation of genetic and epigenetic alterations. Repeated cycles of injury and regeneration render hepatocytes more susceptible to the detrimental effects of mutagenic stimuli [6,7]. After acquiring the requisite number of genetic and epigenetic alterations, the formation of dysplastic foci and nodules occurs, eventually evolving into HCC [8]. HCC has a complex mutational landscape involving major pathways, including genes in Wnt/beta-catenin, TP53, telomere maintenance, oxidative stress response, epigenetic remodeling, and PI3K/Akt/mTOR signaling pathways [9]. Despite the emerging breadth of molecular inhibitors alongside the FDA-approved chemotherapeutics, the survival rate of HCC patients remains low. Owing to the genomic complexity of HCC, the development of effective therapeutic regimens remains a daunting task. 

The central dogma of molecular biology proposed by Francis Crick advocated that the only role of RNA was to encode proteins [10]. With time, the advancement in high-throughput transcriptomic studies has given an unprecedented picture of the fascinating complexity of the human transcriptome. It is now evident that while less than 2% of the human genome encodes proteins, more than 70% is actively transcribed into a variety of non-coding RNAs, including short nuclear and small nucleolar RNAs (snRNAs and snoRNAs), piwi-interacting RNAs (pi-RNAs), microRNAs (miRNAs), long noncoding RNAs (lncRNAs) and circular RNAs (circRNAs) [11]. Numerous studies have revealed a group of small regulatory RNAs, such as miRNAs which fine-tune the key biological processes via regulating messenger RNA (mRNA) translation. Nevertheless, miRNAs are just a small facet of noncoding RNAs scratching only the surface of the RNA world. 

Long noncoding RNAs (lncRNAs), once depicted as ‘the dark matter of the genome’, have drawn increasing attention. LncRNAs are transcripts longer than 200 nucleotides in length. They are expressed in a highly tissue-specific manner. Similar to mRNAs, most lncRNAs are transcribed by RNA polymerase II, 5′-capped, spliced and polyadenylated [12]. Despite lacking protein-coding capacity, multiple lines of evidence have highlighted the important roles of lncRNAs in human cancers [12]. Taking up the majority of the mammalian transcriptome, lncRNAs have been found to fulfill a wide array of regulatory roles at every stage of gene expression. Depending on the subcellular localization, lncRNAs may exert their roles through the crosstalk with RNA, DNA, and proteins. To date, dozens of lncRNAs have been inextricably linked to HCC progression [13], suggesting that lncRNAs are potential therapeutic targets. 

In this review, we present an overview of the current knowledge of lncRNAs in HCC progression and scrutinize their modes of action in cancer phenotypes. We also discuss the potential applications of lncRNAs as prognostic biomarkers and therapeutic targets for HCC patients, as well as future research directions to fully understand the diverse mechanisms of lncRNAs in HCC. 

## 2. Classification of lncRNAs

With the advancement of sequencing technologies, the number of annotated lncRNAs has skyrocketed in recent years. Currently, more than 50,000 lncRNAs have been identified, with approximately 48,000 lncRNA transcripts curated in the Encyclopedia of DNA Elements (ENCODE) Project Consortium (GENCODE release 36) and 27,919 human lncRNAs with high-confidence 5′ ends in Functional Annotation of Mammalian Genomes (FANTOM5). LncRNAs are highly heterogeneous. In contrast to small RNAs, it is challenging to classify lncRNAs because of poor sequence conservation, differential subcellular localization, structural variation, and diverse molecular actions [14]. Depending on their genomic locations relative to protein-coding genes, as well as their origins and directions of transcription, lncRNAs can be classified into six categories: antisense lncRNA, which is transcribed oppositely to a protein-coding gene; bidirectional lncRNA, whose promoter is close to its adjacent protein-coding gene; intronic lncRNA, which lies within an intronic region of a protein-coding gene; intergenic lncRNA, which does not overlap with the sequence of a protein-coding gene; overlapping lncRNA, which encompasses the sequence of a protein-coding gene; and enhancer RNA, which arises from enhancer regions [15]. 

## 3. Roles of Oncogenic and Tumor-Suppressive lncRNAs in HCC

**Figure 1:**
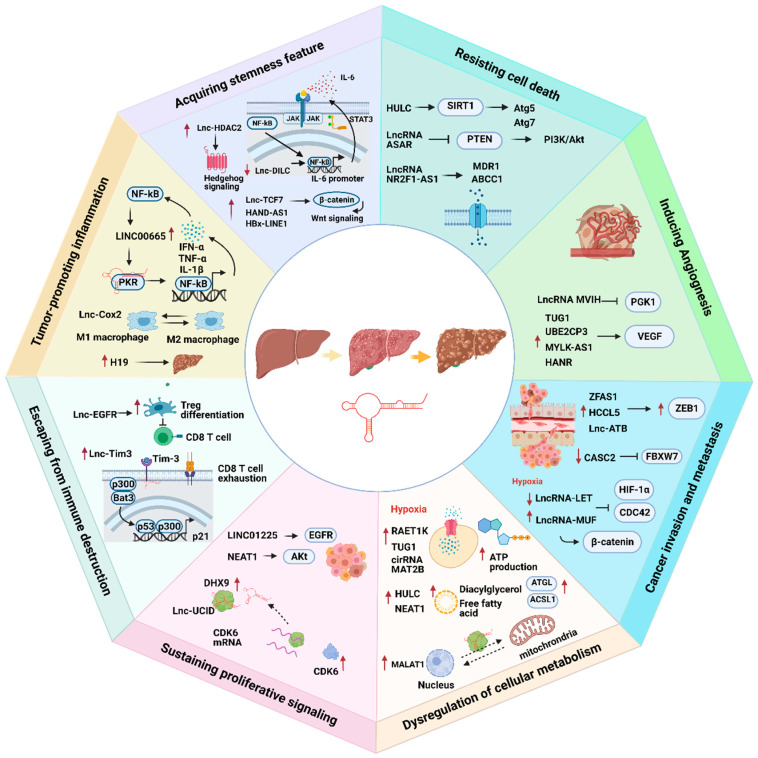
The association of lncRNAs with eight cancer hallmarks in hepatocellular carcinoma (HCC). Red arrows represent up- or downregulation of lncRNAs. Black arrows represent the flow of biological processes.

LncRNAs play an indispensable role in promoting HCC progression (Figure 1).

### 3.1. Sustaining Proliferative Signaling

Cancer cells escape from the normal balance of cell growth by sustaining proliferative signaling [16]. The dysregulation of receptor tyrosine kinases (RTK) such as epithelial growth factor receptor (EGFR), c-Met, and HER2 often occurs in HCC, which subsequently contributes to constant activation and autophosphorylation of a wide range of downstream signaling pathways such as RAS/MAPK, PI3K/AKT, and JAK2/STAT signaling, thereby driving cell proliferation in HCC [16]. Several lncRNAs have been shown to promote cell proliferation by regulating RTK-related gene expression, such as NEAT1 [17]. Furthermore, the overexpression of LINC01225 promotes HCC proliferation by binding to EGFR, which subsequently increases the protein level of EGFR and activates a network of intracellular transduction of proliferative signals [18]. In addition to the dysregulation of RTK-signaling pathways, cancer cells acquire the capability to avert cell cycle arrest, consequently promoting HCC proliferation. There are several lines of evidence to support the notion that protein levels and kinase activities of CDK4, CDK6, and Cyclin D1, the key drivers of G1/S transition leading to early activation of DNA replication, are significantly higher in advanced HCC [19]. A recent report postulated that an lncRNA named lnc-UCID promotes G1/S transition and hepatoma growth by sequestering DHX9 from CDK6 3′UTR, thereby maintaining CDK6 mRNA stability in HCC [20].

### 3.2. Dysregulating Cellular Energies

To fulfill the overwhelming demand for the high rate of proliferation, cancer cells accumulate metabolic alterations to acquire necessary nutrients from a nutrient-deprived environment for the creation of new biomass [21]. High glycolytic rate confers adaptive advantages to cancer cells for fueling ATP production [21]. Under low oxygen conditions, hypoxia-inducible factor 1α (HIF-1α) activates the transcription of lncRNA RAET1K to exaggerate the rate of glucose uptake [22]. In addition, recent studies showed that the overexpression of TUG1 and circular RNA MAT2B enhances glycolysis rate through transcriptional and translational regulation of glycolytic enzymes [23,24].

Cancer cells always carve for more glucose from the surrounding tumor microenvironment to support the high energy demand. When blood glucose levels drop, glucogenesis, a reverse process of glycolysis, is initiated to regenerate glucose from the liver and secrete it into the bloodstream [21]. However, such energy consumption would be an obstacle to sustaining cancer cell viability. A recent study showed that MALAT1 represses the activity of gluconeogenic enzymes and upregulates the expression of glycolytic genes by enhancing the translation of metabolic transcription factor TCF7L2 [25]. In addition to the dramatically increased rate of glycolysis, HCC has been associated with the dysregulation of lipid metabolism. A high rate of lipid uptake and de novo lipid synthesis facilitates the formation of lipid bilayers, thereby accommodating the high rate of proliferation of HCC cells. A recent study documented the ability of HULC in modulating abnormal lipid metabolism. HULC stimulates the accumulation of intracellular triglycerides and cholesterol through overexpressing acetyl-CoA synthetase. Cholesterol addiction promotes a positive feedback loop to activate HULC expression, thereby enhancing the proliferation of HCC cells [26]. 

Another study found that NEAT1 disrupts the lipolysis of hepatoma cells via a lipolytic enzyme adipose triglyceride lipase (ATGL), which ultimately maintains a high level of free fatty acid and diacylglycerol that favors HCC cell growth [27]. As a bioenergetic and signaling hub, mitochondria utilize substrates from the cytoplasm to fuel oxidative mitochondrial metabolism and synthesize an array of biomolecules and NADPH for redox homeostasis [28]. In some cases, lncRNAs have been associated with mitochondrial dysfunction. As a nuclear lncRNA, MALAT1 hitches a ride with RNA transporters into mitochondria where MALAT1 interacts with mitochondrial DNA to regulate mitochondrial metabolism of HCC cells [29]. Nevertheless, it remains unclear what factors govern the trafficking of lncRNA from the nucleus to mitochondria.

### 3.3. Cancer Invasion and Metastasis

To migrate from the original tumor site, disseminate throughout the body, and develop a new tumor site, cancer cells undergo epithelial-to-mesenchymal (EMT) progression that induces a shift in the expression of signaling molecules [30]. In addition to the well-characterized lncRNAs namely MALAT1 and HOTAIR, several lncRNAs have been reported to be critical drivers for HCC progression. For example, ZFAS1, lnc-ATB, and HCCL5 promote the upregulation of ZEB1 [31,32,33]. On the other hand, down-regulation of lncRNA CASC2 relieves miR-367 from RNA sponging. Activation of miR-367 in turn suppresses the tumor-suppressor gene FBXW7 to promote metastasis in HCC [34]. Moreover, hypoxia induces low expression of lncRNA-LET in HCC. Down-regulation of lncRNA-LET stabilizes HIF-1α and CDC42 mRNA, leading to hypoxia-induced cancer cell invasion [35]. 

### 3.4. Inducing Angiogenesis

In light of the increasing demand for oxygen and nutrients, cancer cells produce many pro-angiogenic factors to stimulate the sprouting of new blood vessels from pre-existing blood vessels [36]. Specifically, vascular endothelial growth factor (VEGF) is significantly upregulated in most cancers. Factors including hypoxic conditions, ROS production, and secretion of growth factors and cytokines greatly contribute to the upregulation of VEGF. Not surprisingly, some lncRNAs such as UBE2CP3 and MYLK-AS1 have been shown to modulate the process of angiogenesis through activating the VEGF signaling pathway [37,38]. Furthermore, lncRNA MVIH, whose expression level predicts poor outcome in HCC patients, activates tumor-inducing angiogenesis by inhibiting the secretion of a glycolytic enzyme phosphoglycerate kinase (PGK1) [39]. Lymphatic vasculature facilitates tissue fluid absorption and trafficking of immune cells during inflammation. Unfortunately, under oncogenic stress, the generation of new lymphatic vessels facilitates tumor cell dissemination [40]. lncRNA HANR sequesters the tumor-suppressor miR-296, which in turn increases VEGF level to promote lymphangiogenesis [41]. 

### 3.5. Resisting Cell Death

To ensure constitutive cell growth, cancer cells must circumvent programmed cell death in response to metabolic and therapeutic stress. Apoptosis is a cellular suicide program that promotes anti-tumorigenic effects [42]. LncRNAs are capable of influencing drug resistance through dysregulating apoptotic pathways. In an attempt to study the underlying causes of chemoresistance of HCC cells, Xie et al. found that lncRNA PDIA3P1 is upregulated following the treatment of DNA-damaging chemotherapeutic agents. Mechanistically, PDIA3P1 relieves the repression of miR-125 on TRAF6, leading to the activation of the nuclear factor kappa B (NF-kB) pathway that promotes anti-apoptotic effects [43]. 

Autophagy is characterized by the degradation of organelles of damaged cells as a means of recycling nutrients and sustaining cellular metabolism [44]. Cancer cells can exploit protective autophagy to alleviate the cytotoxicity of chemotherapeutic drugs. Accumulating evidence has revealed several lncRNAs involving in modulating the dynamics of autophagic-induced drug resistance in HCC. For example, conventional chemotherapy induces the overexpression of HULC, which elicits autophagy by enhancing the protein level of SIRT1 and activating autophagy-related genes namely Atg5 and Atg7, thereby protecting HCC cells from chemotherapeutic stress-induced cell death [45]. Autophagy contributes to anoikis, a form of apoptosis that is demarcated by cell detachment from the extracellular matrix [44]. Cancer cells overcome anoikis stress by activating PI3K/Akt pathway. A recent study showed that lnc-ARSR contributes to doxorubicin resistance by activating PI3K/Akt pathway following the inactivation of PTEN [46]. Multidrug resistance (MDR) is a bane of HCC therapy. In addition to dysregulating the programmed apoptotic and autophagic pathways, MDR can be accomplished by increasing drug efflux pumps, including ATP-binding cassette (ABC) drug efflux. LncRNA NR2F1-AS1 promotes oxaliplatin resistance in HCC cells by augmenting the expressions of drug resistance-related genes including MDR1 and ABCC1 [47]. 

### 3.6. Acquiring Stemness Feature

The heterogeneity of HCC arises from the hierarchical organization of tumor cells where a minority population of cancer stem cells (CSCs) residing in the tumor bulk is responsible for maintaining the tumor architecture [48]. Hence, CSCs are the major culprits leading to tumor recurrence. The dysregulation of multiple signaling pathways endows CSCs with the capacity for self-renewal, drug resistance, cancer metastasis, and immune evasion. Several key signaling pathways, such as Hippo-YAP, Wnt/beta-catenin, transforming growth factor-beta (TGF-beta), Notch, and Hedgehog pathways, appear critical for regulating the niche of CSCs [48]. In HCC, the side-population of CSC has been demarcated by distinct cell surface markers (e.g., CD133, CD24, CD90, and epithelial cell adhesion molecule (EpCAM) [49]. A significant body of studies has deciphered the underlying mechanism of lncRNAs in regulating HCC CSCs. LncHDAC2 is highly expressed in CD133+ HCC cells, and that high expression promotes self-renewal of liver CSCs by activating Hedgehog signaling cascades [50]. Numerous self-renewing growth factors and cytokines have been implicated in the maintenance of CSCs, with the activation of several critical transcription factors such as NF-kB and STAT3 [51,52]. Intriguingly, a previous study opined that Inc-DILC serves as a tumor suppressor lncRNAs to inhibit liver CSC expansion through the suppression of IL-6 autocrine signaling [53]. 

### 3.7. Tumor-Promoting Inflammation

Hepatitis viral infection evokes an inflammatory response involving extensive production of cytokines and immune cell infiltration, thereby creating a tumor microenvironment that favors the development of HCC [54]. Production of inflammatory cytokines activates key transcription factors such as NF-kB to induce the transcription of genes promoting cell proliferation and survival [54]. The activation of NF-kB in HCC cells induces the upregulation of LINC00665, which exerts its oncogenic function through promoting the activation and stability of double-stranded RNA-activated protein kinase, resulting in positive feedback on the NF-kB signaling to sustain tumor-associated inflammation [55]. Macrophages exhibit a high degree of dynamic polarization. Ye et al. discovered the role of lnc-Cox2 in macrophage polarization where knockdown of lnc-Cox2 compromises the tumoricidal ability of M1, suppresses the tumor growth, and activates M2 macrophages, indicating the suppressive role of lnc-Cox2 in tumor-inflammatory response [56]. More strikingly, knockout mice models have corroborated the roles of lncRNAs in chronic inflammation-mediated hepatocarcinogenesis. By using the Mdr2-Knockout mouse model to mimic the process of inflammatory-induced HCC development, Gamaev et al. found that high expression of lncRNA H19 further aggravates HCC tumorigenesis. Contrary results were observed in the double KO mouse model (Mdr2-KO/H19-KO) where the development of HCC is impeded in the absence of H19 [57]. 

### 3.8. Escaping from Immune Destruction

Tumor cells adopt different strategies to circumvent immune detection [58]. At the early stage of tumorigenesis, natural killer cells and effector T cells eliminate more immunogenic cancer cells. Once the selected cancer cells escape from tumoricidal immunity, they undergo additional immune-tolerance mechanisms orchestrated by regulatory T cells (Tregs) and tumor-associated macrophages (TAMs) [58]. Accumulating evidence has revealed the crosstalk between lncRNAs and immune cells to modulate the tolerogenic immune response. In HCC, lnc-EGFR sustains the activation of EGFR signaling cascades to promote Treg differentiation and suppress the activity of cytotoxic T lymphocytes [59]. In addition, Lnc-Tim3 is upregulated in tumor-infiltration CD8 T cells of HCC patients. Mechanistically, Lnc-Tim3 promotes CD8 T cell exhaustion via binding to Tim-3, which triggers nuclear localization of Bat3 to enhance p300/p53/p21-mediated cell cycle arrest and compromise the anti-tumor immunity [60]. In contrast to Lnc-EGFR and Lnc-Tim-3, FENDRR suppresses the Treg-mediated immune escape, outlining the potential role of FENDRR as an alternative immune-based treatment strategy [61]. 

## 4. Functional Mechanism of lncRNAs

**Figure 2:**
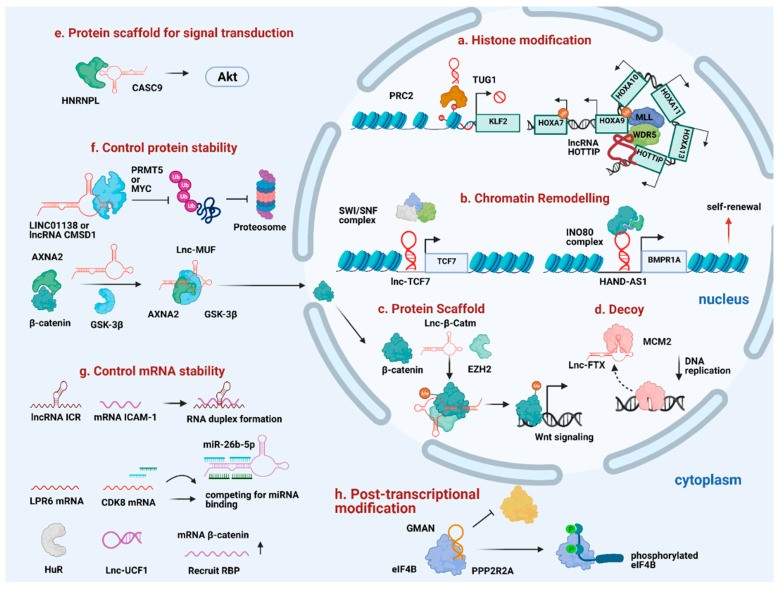
Diverse mechanism of lncRNAs in promoting HCC progression. (**a**) TUG1 and HOTTIP act as guide to recruit histone modifiers to the specific loci. (**b**) Lnc-TCF7 and HAND-AS1 recruit SWI/SNF complex to specific gene promoters to promote self-renewal. (**c**) Lnc-β-Catm reinforces protein-protein interaction and controls protein stability. (**d**) Lnc-FTX serves as a molecular decoy to move MCM2 away from the chromatin. (**e**) Cytoplasmic lncRNAs like CASC9 interacts with HNRNPL to promote HCC progression. (**f**) LINC01138, CMSD1, and Lnc-MUF interact with protein partners to control protein stability. (**g**) Some lncRNAs control mRNA stability through forming RNA duplex with target mRNA, acting as competitive sponge for miRNA binding or recruit RNA-binding protein to stabilize target mRNA stability. (**h**) GMAN regulates post-transcriptional modification by disrupting the interaction between target protein and protein-modifying enzyme.

Depending on the subcellular localization, lncRNAs have profound impacts on transcriptional and post-transcriptional control. While nuclear lncRNAs act as ‘RNA bridges’ to facilitate the interaction between chromatin-modifying complexes and chromatin, as well as promote higher-order chromatin architecture, cytoplasmic lncRNAs act as post-transcriptional regulators to regulate mRNA decay and protein translation or act as ‘sponge’ for miRNA sequestration. Considering the unique expression and exquisite tissue-specificity of lncRNAs, researchers have been exploring the functional crosstalk between lncRNAs and epigenetic machinery in promoting HCC progression (Figure 2).

### 4.1. Nuclear-Specific lncRNAs in Transcriptional Control

If functional, lncRNAs that are enriched at their own sites of transcription are expected to be involved in chromatin modification processes. Compiling evidence supports the roles of lncRNAs in regulating transcriptional activity of local or distal genes using different approaches, such as modulating the activities of epigenetic regulators, direct interaction between lncRNAs and DNA, as well as facilitating enhancer-promoter interaction.

#### 4.1.1. Chromatin Remodeling and Histone Modification

Aberrant histone modification profiles are frequently observed during HCC progression owing to the dysregulation of epigenetic regulators [7]. Notably, various transcription repressive histone methyltransferases including EZH2, SETDB1, and G9a are frequently deregulated in HCC, resulting in the epigenetic silencing of tumor-suppressor genes [62,63,64]. Supporting the contention that lncRNAs interface with these epigenetic modifiers to influence gene transcription, a recent study utilized multiple genetic and chemical approaches to underscore the roles of RNA in modulating the activity of polycomb repressive complex 2 (PRC2) [65]. The perturbation of RNA-PRC2 interaction results in a substantial loss of PRC2 at the genomic targets, consequently promoting the pre-mature expression of repressive genes that lead to functional abnormalities of the cardiomyocytes. In HCC, TUG1 facilitates the recruitment of PRC2 to deposit the H3K27me3 silencing mark at the promoter of KLF2 and suppress KLF2 expression [66]. Beyond repressive histone methyltransferases, several lncRNAs have played a role in trafficking transcription activators to the specific loci. This mechanism is exemplified by HOTTIP, where it interacts with the WDR5/MLL complex and localizes it to the proximity of the HOXA locus, leading to transcriptional activation of HOXA genes, and thus, hepatocarcinogenesis [67]. Another study demonstrated the *cis*-regulatory role of 91H on its neighboring gene IGF2 through recruiting RBBP5 [68]. A similar recruiting mechanism has been observed in lncAKHE, which cooperates with transcription activator YEATS4 to enhance NOTCH2 signaling in HCC [69]. 

HCC displays distinct DNA methylation signatures associated with HCC grades and patient survival [70,71]. A few studies have inferred the roles of lncRNAs in modulating the activity of DNA methyltransferases (DNMTs), resulting in the alteration of DNA methylation. It was reported that LncRNA34a recruits DNMT3a to promote DNA methylation activity on miR-34a promoter in HCC. This recruitment promotes lnc-34a mediated transcriptional silencing of miR-34a [72]. 

Mutations in genes encoding for components of ATP-dependent chromatin remodeling complexes such as switching defective/sucrose nonfermenting (SWI/SNF) complex and INO80 exhibit significant impacts on HCC progression [73]. Recent studies have shown the capacity of lncRNAs to regulate gene expression and remodel chromatin accessibility through interacting with SWI/SNF complexes [74]. For example, lncTCF7 promotes transcriptional activation of its neighboring gene TCF7 by recruiting the SWI/SNF complex to the promoter of TCF7, which in turn triggers the activation of the Wnt signaling pathway to maintain the self-renewal of HCC CSCs [75]. Another study revealed the functional interdependence between lncBRM and a subunit of SWI/SNF complex BRM, where lncBRM physically associates with BRM to initiate the activation of YAP1 signaling and sustain the self-renewal of HCC CSCs [76]. Besides SWI/SNF complex, lncRNA HAND2-AS1 recruits INO80 to the promoter of BMPR1A, thereby inducing the expression of BMPR1A to promote liver CSCs self-renewal [77]. Together, these findings highlight the functional crosstalk between lncRNAs and epigenetic modifiers in controlling the transcriptional output of target genes. 

#### 4.1.2. Scaffolds for Protein-Interacting Partners/Transcription Factors

Although proteins can serve as scaffolds for other proteins, RNA-protein scaffold is more cost-effective, as an RNA molecule comprising 100 nt can capture more than five proteins simultaneously [78]. Several studies have suggested that lncRNAs interact with proteins to regulate a joint set of target gene transcription, thereby promoting HCC tumorigenesis [79,80]. Indeed, lncRNAs have been shown to reinforce protein-protein interaction. Zhu et al. observed through domain mapping that a lncRNA Lnc-β-Catm physically interacts with β-catenin and EZH2. EZH2 methylates β-catenin to suppress the ubiquitination of β-catenin, thereby allowing β-catenin to activate Wnt-signaling to sustain the self-renewal of HCC CSCs [81]. 

While the lncRNAs cited above exert oncogenic roles in HCC, other lncRNAs have been reported to suppress tumor growth through co-operating with other proteins in HCC [82,83]. For instance, downregulation of TCAM1P-004 and RP11-598D14.1 abolishes their tumor-suppressive effects by disrupting their interaction with their protein partners IGF2BP1, HIST1H1C, and STAU1, consequently repressing the expression of apoptotic factor DDI3 in HCC [83]. Some lncRNAs have been reported to act not by recruiting but rather by sequestering proteins from the chromatin to regulate gene transcription. For example, lnc-FTX binds DNA replication licensing factor MCM2, causing a decrease in chromatin-bound MCM2, thereby impeding DNA replication and inhibiting HCC growth [82]. Nevertheless, many proteins lack canonical RNA binding domains [65]. Whether these proteins can identify specific RNA sequences or structure or whether they bind RNA promiscuously remains unclear. 

#### 4.1.3. Modulating Chromatin Architecture

While the interplay with epigenetic machinery enables lncRNAs to recognize gene loci, several lines of evidence have pointed to the connection of lncRNAs with chromatin architecture. One of the mechanisms by which lncRNAs directly bind to genomic DNA is R-loop formation. For example, an antisense lncRNA VIM-AS1 forms an R-loop around the VIM promoter to induce chromatin opening that favors NF-kB binding and promotes VIM transcription [84]. Another way of direct lncRNA-chromatin interaction is the formation of RNA-DNA triplex that accelerates transcriptional induction [85]. A previous study demonstrated an interesting model where an antisense lncRNA Khps1 forms RNA/DNA triplex around the SPHK1 promoter to trigger an open chromatin structure and facilitate the recruitment of CBP/p300, thereby driving transcriptional activation of SPHK1 [86]. Furthermore, the 3D architecture of the chromosome can allow lncRNAs to spread their effects over distal regions along the same chromosome [87]. For example, CCAT1, which is transcribed from super-enhancer upstream of MYC, promotes transcriptional activation of MYC by promoting chromatin looping that places CCAT1 in the proximity to MYC with the recruitment of CTCF in human colorectal cancer [88]. These findings collectively highlight the indispensable role of lncRNAs in modulating chromatin architecture to promote target gene transcription. However, much remains to be determined regarding what drives lncRNAs to modulate chromatin architecture and whether such transcriptional regulation is involved in promoting HCC progression. 

### 4.2. Cytoplasmic-Specific lncRNAs in Post-Transcriptional Control

LncRNAs are not restricted to the control of the localization of proteins on the chromatin. Many lncRNAs are exported to the cytoplasm where they modulate two cytoplasmic processes that have a profound impact on protein translation—mRNA turnover and translation. 

#### 4.2.1. mRNA Stability

LncRNAs can regulate mRNA dynamics at a post-transcriptional level through (1) miRNA sponge, (2) mRNA interaction, and (3) recruitment of RBPs. Sequence-specific interaction renders lncRNAs to fine-tune gene expression that favors HCC tumorigenesis by modulating mRNA stability and miRNA availability. LncRNA-mRNA complex can prevent microRNAs from binding the target mRNAs, thereby reversing the suppressive effect of miRNAs on mRNA targets [89]. For instance, lncRNA DANCR occupies the miRNA binding site on CTNNB1 3′UTR, which increases the population of tumor cells with stemness features [90]. In addition, the lncRNA-mRNA complex can increase the stability of mRNA. For example, ICAM-1-related noncoding RNA (ICR) increases the stability of its target mRNA ICAM-1 through RNA duplex formation to maintain the stem cell properties of ICAM+HCC CSCs [91]. Some lncRNAs function as competing for endogenous RNAs (ceRNAs) to compete for miRNA binding, consequently reducing miRNA action on mRNA targets [33,92,93,94,95]. An example of this lncRNA-miRNA interplay is DLGAP1-AS1, which sequesters HCC-inhibitory miRNAs, miR-26a-5p and miR-26b-5p, and thus, enhances IL-6 level to activate JAK2/STAT3 signaling during HCC progression [93]. 

Besides miRNA sponge, some lncRNAs can modulate mRNA expression by recruiting RBPs. For example, lncRNA UFC1 binds with mRNA stabilizing protein HuR to stabilize and increase the level of β-catenin mRNA [96]. Moreover, lncRNAs can form a complex with RBPs to mediate transcriptional repression. It has been reported that lncRNA miR-503HG interacts with heterogeneous nuclear ribonucleoprotein A2/B1 (HNRNPA2B1) to promote its degradation, which in turn reduces the stability of p53 and p65 mRNA and eventually inhibits NF-kB signaling pathway and EMT process in HCC [97]. 

#### 4.2.2. Protein Stability

Compiling evidence has indicated that lncRNAs can regulate protein stability through controlling ubiquitin or proteasome machinery. Some lncRNAs exert their oncogenic functions through stabilizing oncoproteins in HCC [98,99,100]. For example, lncRNA CMSD1 specifically binds and stabilizes MYC protein to protect it from ubiquitin-proteasome degradation, thereby promoting hepatocarcinogenesis [100]. Likewise, LINC01138 promotes HCC tumorigenesis by enhancing the stability of arginine methyltransferase (PRMT5) and that LINC01138 function depends on the presence of PRMT5 [99]. In contrast, a tumor-suppressive lncRNA RP11-286H15.1 binds to poly (A) binding protein 4 (PABPC4) to promote its ubiquitination [101]. Similarly, lncRNA PSTAR inhibits HCC tumorigenicity by enhancing the SUMOylation modification of heterogeneous nuclear ribonucleoprotein K (hnRNP-K), which further strengthens the interaction between hnRNP-K and p53, leading to the transactivation of p53 [102].

#### 4.2.3. Signal Transduction

Some lncRNAs work with RBPs to promote signal transduction in the cytoplasm. For example, CASC9 forms a functional cytoplasmic complex with RBP heterogeneous nuclear ribonucleoprotein L (HNRNPL), consequently activating the AKT pathway-associated signaling molecules to promote hepatocarcinogenesis [103]. Furthermore, some lncRNAs control the subcellular mobilization of proteins. For example, lnc-MUF promotes the interaction of AXNA2 and GSK-3β, whereby AXNA2 alters the subcellular localization of β-catenin to activate Wnt signaling cascades by protecting β-catenin from GSK-3β-mediated degradation [80]. In some cases, lncRNAs interact with proteins and modulate signal transduction pathways by modifying their phosphorylation status. For example, HNF1A-AS1 binds to the C-terminal of Src homology region 2 (SH2) domain-containing phosphatase 1 (SHP-1) protein and increases the phosphatase activity of SHP-1, which subsequently promotes anti-tumor effects of HNF-1α and HNF1A-AS1 [104]. Interestingly, some lncRNAs modulate protein translation. LncRNA GMAN was found to interact with eukaryotic translation initiation factor 4B (eIF4B) and promote its phosphorylation by preventing dephosphorization of another protein mediator phosphatase 2A subunit B (PPP2R2A). These changes subsequently increase the expression of anti-apoptosis-related protein expression, thereby promoting HCC tumorigenesis [105]. 

## 5. From Cancer Hallmarks to Clinical Utility

Ultrasound imaging and alpha-fetoprotein (AFP) measurement are the current surveillance tools for the early detection of HCC [106]. However, these detection approaches have unconquerable limitations. While ultrasound imaging detects early-stage HCC with only 47% sensitivity, the sensitivity and specificity of AFP for initial HCC diagnosis is low, indicating an urgent need for novel biomarkers in the hope of enhancing early detection of HCC [107]. The crucial roles of lncRNAs in diverse cellular processes render them appealing candidates for the diagnosis and treatment of HCC. The exquisite tissue-specificity of lncRNAs can help classify different subclasses of tumors or even help predict different clinical outcomes. This realization has spurred further exploration of the advantages of quantifying lncRNA expression in tumor biopsies and plasma. Some lncRNAs are highly specific in tumor patients compared with healthy counterparts, and their striking expression patterns in tumors make them detectable in body fluids such as plasma, blood, and saliva, indicating the potential as non-invasive diagnostic biomarkers (Table 1). For example, HULC, whose expression predicts poor clinical outcome for HCC patients, is detected in the blood of HCC patients and its corresponding tumor tissues by the conventional PCR method. Other lncRNAs such as MALAT1, LINC00152, RP11-160H22.5, Lnc-PCDH9-13:1, and XLOC014172 have been reported to be circulating lncRNAs [108,109,110,111]. In addition, the collection of exosomal lncRNAs becomes a new source of biomarkers for non-invasive cancer diagnosis. As a signal molecule of cell-to-cell communication, exosomes containing all biological components provide a more comprehensive resolution of cancer heterogeneity [112]. Exosomes include tumor-specific lncRNAs that are protected from enzymatic degradation in body fluid, rendering them attractive alternatives for non-invasive biomarkers. Extracellular vesicle long RNA sequencing (exLR-seq) detects more than 10,000 exosomal long RNAs, some of which are HCC-overexpressing lncRNAs with functional implications [113]. In addition, some exosome-derived lncRNAs, such as LINC00853, lncRNA-ATB, LINC00511 are associated with prognostic factors for HCC patients [108,113,114,115], indicating potential diagnostic biomarker for early HCC [114]. Some lncRNAs such as lncRNA-VLDLR and lncRNA-ROR have been implicated in modulating hepatoma cellular responses to Sorafenib through extracellular vesicle-mediated intracellular signaling in HCC. These findings open an avenue for direct precision medicine [116,117]. 

As discussed above, lncRNAs regulate specific facets of cellular networks, suggesting that they are desirable for potential therapeutic regimens. Considering the undesirable toxic effects of direct protein target inhibition, drugs targeting lncRNAs would be a desirable strategy for the inhibition of protein activity. The most promising attempt of lncRNA inhibition therapy is based on the use of antisense oligonucleotides (ASOs), which have been reported to have excellent efficacy in reducing lncRNA levels, especially nuclear-specific lncRNAs, as exemplified by the success of targeting LINC00210 that impairs self-renewal of CSCs in vitro in HCC and targeting lncRNA SCAT7 in vivo in lung cancer [118,119]. Given the interplay between lncRNAs and epigenetic modifiers such as PRC2, researchers are developing synthetic molecules that occupy the binding sites where target lncRNAs and PRC2 interact to de-repress PRC2-target genes [120]. Besides blocking the interaction sites, investigators are exploring synthetic ncRNAs molecules to establish tumor-suppressive functions [121]. The development of lncRNA-based inhibitors helps reshape the drug discovery landscape; however, a few challenges regarding the delivery system, as well as adverse effects remain to be addressed.

**Table 1:** Examples of lncRNA markers with potential prognostic and diagnostic values for HCC patients.

Examples of LncRNAs	Source of Biomarkers	Method of Analysis	Roles in HCC	Modes of Action	Biological Functions	Reference
HULC	Blood	qRT-PCR	Oncogene	miRNA sponge; Stabilizing SIRT1	Promote metastasis and chemotherapy resistance	[45,109]
MALAT1	Plasma	qRT-PCR	Oncogene	SRSF1 upregulation and mTOR activation	Promote metastasis and tumorigenesis	[110,122]
LINC00152	Plasma	qRT-PCR	Oncogene	Upregulate the expression of CCND1 through miRNA sponge	Promote cell cycle progression	[115,123]
LINC00511	Serum-derived exosomes	qRT-PCR	Oncogene	Upregulate EYA1 expression through miRNA sponge	Promote cell proliferation and migration	[108,124]
LINC00161	Serum-derived exosomes	qRT-PCR	Oncogene	Activate ROCK2 signaling pathway through miRNA sponge	Promote angiogenesis and metastasis	[125]
LncRNA-ATB	Serum-derived exosomes	qRT-PCR	Oncogene	Upregulate ZEB1 expression through miRNA sponge	Promote metastasis	[33,108]
ZFAS1	Plasma	qRT-PCR	Oncogene	Upregulate ZEB1 expression through miRNA sponge	Promote metastasis	[31,126]

## 6. Future Perspectives and Challenges

Taking the advantage of new technologies, we have witnessed remarkable progress in discovering the ‘dark matter’ of the genome. As indispensable ‘game players’ in the genome, lncRNAs interface with epigenetic factors, transcription factors, proteins, and nucleic acids to temporally and spatially modulate their activities, collectively shaping the outcomes of crucial cellular processes. As discussed above, the dysregulation of lncRNAs contributes to HCC cancer phenotypes. Hence, screening the abundance of oncogenic lncRNAs in body fluid becomes a promising strategy for biomarker identification. Targeted lncRNA sequencing is superior in detecting an array of potential oncogenic lncRNAs in multiple pairs of human liquid biopsy [127,128]. Given the functional crosstalk between lncRNAs and epigenetic machinery, targeting RNA becomes a new frontier for drug development. While ASOs represent the best oligo-based drug to target lncRNAs, some RNAs have been reported to be rich in potentially druggable structure, holding a promise for the development of small molecules against target lncRNAs [129,130,131]. The structurally conserved lncRNAs are appealing for predicting physical targets and drug selectivity, although more works are necessary to scrutinize the high-order structure of lncRNAs and RNA-ligand interaction. 

Despite the discovery of a large repertoire of annotated lncRNAs, the era of studying the functions of lncRNAs in HCC is just beginning, and we are still far from clinical trials with lncRNA-based therapeutics for HCC patients. LncRNAs possess many features reminiscent of protein-coding genes such as post-transcriptional modifications. Among them, RNA modifications such as 5-methylcytosine (m^5^C) and N6-methyladenosine (m6A) have recently come to the spotlight in lncRNA studies [132,133]. Several studies have reported that m6A frequently regulates the structure, stability, expression, as well as subcellular distribution of lncRNAs [134,135]. In HCC, m6A modification increases the stability of LINC00958, thereby leading to its overexpression and promoting tumor growth in HCC [136]. m6A modification may affect lncRNA secondary structure, thereby dysregulating the interaction between lncRNAs and their protein partners or other RNA species, consequently driving the dysregulation of signaling pathways that favor tumor progression [136]. Nevertheless, only a handful of studies have reported the roles of m6A modification of lncRNAs in promoting HCC progression. Given that most lncRNAs exert their functions through their protein partners, there is a pressing need to study the underlying mechanisms by which m6A modification regulates oncogenic functions of lncRNAs in HCC. 

One of the greatest challenges is to understand how lncRNA sequences and RNA structure reflect their functions, given their limited sequence conservation and noncoding nature [137]. Attempts to identify functional lncRNAs in HCC have been relied on RNA-seq to find out the aberrant expression of lncRNAs between tumor and non-tumor. However, the differential expression patterns of lncRNAs do not necessarily reflect the functions of lncRNAs. Multiple genetic approaches are required to elucidate the functions of lncRNAs, which seem to be daunting to identify the functions of thousands of lncRNAs at once. Over the last five years, Clustered Regulatory Interspaced Short Palindromic Repeat (CRISPR) screening technology has been employed to interrogate the functions of protein-coding genes and lncRNAs associated with screening phenotypes—proliferation and drug resistance [138,139,140]. CRISPR screening not only enables the discovery of novel functional lncRNAs that influence the phenotype of interest but also facilitates the development of lncRNA-based therapeutic targets across a broad spectrum of human diseases. 

In summary, the dysregulation of lncRNAs is closely associated with HCC progression. However, the field is riddled with many unresolved questions. Future research efforts should go beyond the descriptive identification of differentially expressed lncRNAs and focus on the functions and molecular modalities of lncRNAs in promoting HCC progression, which are extremely necessary for the development of lncRNA-based biomarkers and therapeutics with high specificity and sensitivity.

## Data Availability

Not applicable.

## Abbreviations

ABC	ATP-binding cassette transporters
Akt	Protein kinase B
ASO	Antisense Oligonucleotides
ATGL	Adipose triglyceride lipase
AUG5	Autophagy related 5
AXAN2	Annexin A
CDK4	Cyclin-dependent kinase 4
CircRNAs	Circular RNAs
CRISPR	Clustered Regularly Interspaced Short Palindromic Repeats
CSC	Cancer stem cells
CTCF	CCCTC-binding factor
CTNNB1	Catenin Beta 1
DHX9	DExH-Box Helicase 9
DNMTs	DNA methyltransferases
EGFR	Epithelial growth factor receptor
EMT	Epithelial-to-mesenchymal transition
EpCAM	Epithelial cell adhesion molecule
ExLR-seq	Extracellular vesicle long RNA sequencing
EZH2	Enhancer of zeste homolog 2
G9a	Euchromatic histone-lysine N-methyltransferase 2
GSK-3β	Glycogen synthase kinase 3
H3K27me3	tri-methylation of lysine 27 on histone H3
HCC	Hepatocellular carcinoma
HIF-1α	Hypoxia-inducible factor 1α
HNRNPK	Heterogeneous nuclear ribonucleoprotein K
HNRNPA2B1	Heterogeneous nuclear ribonucleoprotein A2/B1
IGF2BP1	Insulin Like Growth Factor 2 MRNA Binding Protein 1
IL-6	Interleukin-6
INO80	*INO80* Complex ATPase Subunit
KLF2	Krüppel-like Factor 2
LncRNAs	Long noncoding RNAs
mTOR	Mechanistic target of rapamycin
MCM2	Minichromosome Maintenance Complex Component 2
M6A	N 6-methyladenosine
MDR	Multidrug resistance
NF-kB	Nuclear factor kappa B
Nt	Nucleotide
PGK1	Phosphoglycerate kinase
PI3K	Phosphoinositide 3-kinases
PRC2	Polycomb Complex 2
PRMT5	Arginine methyltransferase
RBBP5	RB Binding Protein 5
RNP	RNA binding protein
RTK	Receptor tyrosine kinases
SETDB1	SET domain, bifurcate 1
SIRT1	Sirtuin 1
SPHK1	Sphingosine Kinase 1
STAU1	Staufen Double-Stranded RNA Binding Protein 1
SWI/SNF	SWItch/Sucrose Non-Fermentable
TAMs	Tumor-associated macrophages
TERT	Telomerase reverse transcriptase
TGF-β	Transforming growth factor-beta
TRAF	TNF receptor associated factor
Treg	Regulatory T cells
VEGF	Vascular endothelial growth factor
YAP1	Yes-associated protein 1
ZEB1	Zinc finger E-box-binding homeobox 1

## References

[1] Levrero M., Zucman-Rossi J. (2016). Mechanisms of HBV-induced hepatocellular carcinoma. J. Hepatol..

[2] Gomaa A.I., Khan S.A., Toledano M.B., Waked I., Taylor-Robinson S.D. (2008). Hepatocellular carcinoma: Epidemiology, risk factors and pathogenesis. World J. Gastroenterol..

[3] Ozakyol A. (2017). Global epidemiology of hepatocellular carcinoma (HCC epidemiology). J. Gastrointest. Cancer.

[4] Bruix J., Raoul J.L., Sherman M., Mazzaferro V., Bolondi L., Craxi A., Galle P.R., Santoro A., Beaugrand M., Sangiovanni A. (2012). Efficacy and safety of sorafenib in patients with advanced hepatocellular carcinoma: Subanalyses of a phase III trial. J. Hepatol..

[5] Rimassa L., Danesi R., Pressiani T., Merle P. (2019). Management of adverse events associated with tyrosine kinase inhibitors: Improving outcomes for patients with hepatocellular carcinoma. Cancer Treat. Rev..

[6] Alison M.R., Islam S., Lim S. (2009). Stem cells in liver regeneration, fibrosis and cancer: The good, the bad and the ugly. J. Pathol..

[7] Juhling F., Hamdane N., Crouchet E., Li S., El Saghire H., Mukherji A., Fujiwara N., Oudot M.A., Thumann C., Saviano A. (2021). Targeting clinical epigenetic reprogramming for chemoprevention of metabolic and viral hepatocellular carcinoma. Gut.

[8] Ogunwobi O.O., Harricharran T., Huaman J., Galuza A., Odumuwagun O., Tan Y., Ma G.X., Nguyen M.T. (2019). Mechanisms of hepatocellular carcinoma progression. World J. Gastroenterol..

[9] Severi T., van Malenstein H., Verslype C., van Pelt J.F. (2010). Tumor initiation and progression in hepatocellular carcinoma: Risk factors, classification, and therapeutic targets. Acta Pharmacol. Sin..

[10] Cobb M. (2017). 60 years ago, Francis Crick changed the logic of biology. PLoS Biol..

[11] Hombach S., Kretz M. (2016). Non-coding RNAs: Classification, biology and functioning. Adv. Exp. Med. Biol..

[12] Rinn J.L., Chang H.Y. (2012). Genome regulation by long noncoding RNAs. Annu. Rev. Biochem..

[13] Wong C.M., Tsang F.H., Ng I.O. (2018). Non-coding RNAs in hepatocellular carcinoma: Molecular functions and pathological implications. Nat. Rev. Gastroenterol. Hepatol..

[14] Mattick J.S., Rinn J.L. (2015). Discovery and annotation of long noncoding RNAs. Nat. Struct. Mol. Biol.

[15] Cabili M.N., Dunagin M.C., McClanahan P.D., Biaesch A., Padovan-Merhar O., Regev A., Rinn J.L., Raj A. (2015). Localization and abundance analysis of human lncRNAs at single-cell and single-molecule resolution. Genome Biol..

[16] Feitelson M.A., Arzumanyan A., Kulathinal R.J., Blain S.W., Holcombe R.F., Mahajna J., Marino M., Martinez-Chantar M.L., Nawroth R., Sanchez-Garcia I. (2015). Sustained proliferation in cancer: Mechanisms and novel therapeutic targets. Semin. Cancer Biol..

[17] Chen S., Xia X. (2019). Long noncoding RNA NEAT1 suppresses sorafenib sensitivity of hepatocellular carcinoma cells via regulating miR-335-c-Met. J. Cell. Physiol..

[18] Wang X., Zhang W., Tang J., Huang R., Li J., Xu D., Xie Y., Jiang R., Deng L., Zhang X. (2016). LINC01225 promotes occurrence and metastasis of hepatocellular carcinoma in an epidermal growth factor receptor-dependent pathway. Cell Death Dis..

[19] Masaki T., Shiratori Y., Rengifo W., Igarashi K., Yamagata M., Kurokohchi K., Uchida N., Miyauchi Y., Yoshiji H., Watanabe S. (2003). Cyclins and cyclin-dependent kinases: Comparative study of hepatocellular carcinoma versus cirrhosis. Hepatology.

[20] Wang Y.L., Liu J.Y., Yang J.E., Yu X.M., Chen Z.L., Chen Y.J., Kuang M., Zhu Y., Zhuang S.M. (2019). Lnc-UCID promotes G1/S transition and hepatoma growth by preventing DHX9-mediated CDK6 down-regulation. Hepatology.

[21] Pavlova N.N., Thompson C.B. (2016). The emerging hallmarks of cancer metabolism. Cell Metab..

[22] Zhou Y., Huang Y., Hu K., Zhang Z., Yang J., Wang Z. (2020). HIF1A activates the transcription of lncRNA RAET1K to modulate hypoxia-induced glycolysis in hepatocellular carcinoma cells via miR-100-5p. Cell Death Dis..

[23] Lin Y.H., Wu M.H., Huang Y.H., Yeh C.T., Cheng M.L., Chi H.C., Tsai C.Y., Chung I.H., Chen C.Y., Lin K.H. (2018). Taurine up-regulated gene 1 functions as a master regulator to coordinate glycolysis and metastasis in hepatocellular carcinoma. Hepatology.

[24] Li Q., Pan X., Zhu D., Deng Z., Jiang R., Wang X. (2019). Circular RNA MAT2B promotes glycolysis and malignancy of hepatocellular carcinoma through the miR-338-3p/PKM2 axis under hypoxic stress. Hepatology.

[25] Malakar P., Stein I., Saragovi A., Winkler R., Stern-Ginossar N., Berger M., Pikarsky E., Karni R. (2019). Long Noncoding RNA MALAT1 regulates cancer glucose metabolism by enhancing mTOR-mediated translation of TCF7L2. Cancer Res..

[26] Cui M., Xiao Z., Wang Y., Zheng M., Song T., Cai X., Sun B., Ye L., Zhang X. (2015). Long noncoding RNA HULC modulates abnormal lipid metabolism in hepatoma cells through an miR-9-mediated RXRA signaling pathway. Cancer Res..

[27] Liu X., Liang Y., Song R., Yang G., Han J., Lan Y., Pan S., Zhu M., Liu Y., Wang Y. (2018). Long non-coding RNA NEAT1-modulated abnormal lipolysis via ATGL drives hepatocellular carcinoma proliferation. Mol. Cancer.

[28] Zong W.X., Rabinowitz J.D., White E. (2016). Mitochondria and Cancer. Mol. Cell.

[29] Zhao Y., Zhou L., Li H., Sun T., Wen X., Li X., Meng Y., Li Y., Liu M., Liu S. (2020). Nuclear-encoded lncRNA MALAT1 epigenetically controls metabolic reprogramming in HCC cells through the mitophagy pathway. Mol. Ther. Nucleic Acids.

[30] Diepenbruck M., Christofori G. (2016). Epithelial-mesenchymal transition (EMT) and metastasis: Yes, no, maybe?. Curr. Opin. Cell Biol..

[31] Li T., Xie J., Shen C., Cheng D., Shi Y., Wu Z., Deng X., Chen H., Shen B., Peng C. (2015). Amplification of long noncoding RNA ZFAS1 promotes metastasis in hepatocellular carcinoma. Cancer Res..

[32] Peng L., Jiang B., Yuan X., Qiu Y., Peng J., Huang Y., Zhang C., Zhang Y., Lin Z., Li J. (2019). Super-enhancer-associated long noncoding RNA HCCL5 is activated by ZEB1 and Promotes the malignancy of hepatocellular carcinoma. Cancer Res..

[33] Yuan J.H., Yang F., Wang F., Ma J.Z., Guo Y.J., Tao Q.F., Liu F., Pan W., Wang T.T., Zhou C.C. (2014). A long noncoding RNA activated by TGF-beta promotes the invasion-metastasis cascade in hepatocellular carcinoma. Cancer Cell.

[34] Wang Y., Liu Z., Yao B., Li Q., Wang L., Wang C., Dou C., Xu M., Liu Q., Tu K. (2017). Long non-coding RNA CASC2 suppresses epithelial-mesenchymal transition of hepatocellular carcinoma cells through CASC2/miR-367/FBXW7 axis. Mol. Cancer.

[35] Yang F., Huo X.S., Yuan S.X., Zhang L., Zhou W.P., Wang F., Sun S.H. (2013). Repression of the long noncoding RNA-LET by histone deacetylase 3 contributes to hypoxia-mediated metastasis. Mol. Cell.

[36] Nishida N., Yano H., Nishida T., Kamura T., Kojiro M. (2006). Angiogenesis in cancer. Vasc. Health Risk Manag..

[37] Lin J., Cao S., Wang Y., Hu Y., Liu H., Li J., Chen J., Li P., Liu J., Wang Q. (2018). Long non-coding RNA UBE2CP3 enhances HCC cell secretion of VEGFA and promotes angiogenesis by activating ERK1/2/HIF-1alpha/VEGFA signalling in hepatocellular carcinoma. J. Exp. Clin. Cancer Res..

[38] Teng F., Zhang J.X., Chang Q.M., Wu X.B., Tang W.G., Wang J.F., Feng J.F., Zhang Z.P., Hu Z.Q. (2020). LncRNA MYLK-AS1 facilitates tumor progression and angiogenesis by targeting miR-424-5p/E2F7 axis and activating VEGFR-2 signaling pathway in hepatocellular carcinoma. J. Exp. Clin. Cancer Res..

[39] Yuan S.X., Yang F., Yang Y., Tao Q.F., Zhang J., Huang G., Yang Y., Wang R.Y., Yang S., Huo X.S. (2012). Long noncoding RNA associated with microvascular invasion in hepatocellular carcinoma promotes angiogenesis and serves as a predictor for hepatocellular carcinoma patients’ poor recurrence-free survival after hepatectomy. Hepatology.

[40] Christiansen A., Detmar M. (2011). Lymphangiogenesis and cancer. Genes. Cancer.

[41] Shi Y., Yang X., Xue X., Sun D., Cai P., Song Q., Zhang B., Qin L. (2019). HANR promotes lymphangiogenesis of hepatocellular carcinoma via secreting miR-296 exosome and regulating EAG1/VEGFA signaling in HDLEC cells. J. Cell Biochem..

[42] Fernald K., Kurokawa M. (2013). Evading apoptosis in cancer. Trends Cell Biol..

[43] Xie C., Zhang L.Z., Chen Z.L., Zhong W.J., Fang J.H., Zhu Y., Xiao M.H., Guo Z.W., Zhao N., He X. (2020). A hMTR4-PDIA3P1-miR-125/124-TRAF6 regulatory axis and its function in NF kappa B signaling and chemoresistance. Hepatology.

[44] Mathew R., Karantza-Wadsworth V., White E. (2007). Role of autophagy in cancer. Nat. Rev. Cancer.

[45] Xiong H., Ni Z., He J., Jiang S., Li X., He J., Gong W., Zheng L., Chen S., Li B. (2017). LncRNA HULC triggers autophagy via stabilizing Sirt1 and attenuates the chemosensitivity of HCC cells. Oncogene.

[46] Li Y., Ye Y., Feng B., Qi Y. (2017). Long noncoding RNA lncARSR promotes doxorubicin resistance in hepatocellular carcinoma via modulating PTEN-PI3K/Akt pathway. J. Cell Biochem..

[47] Huang H., Chen J., Ding C.M., Jin X., Jia Z.M., Peng J. (2018). LncRNA NR2F1-AS1 regulates hepatocellular carcinoma oxaliplatin resistance by targeting ABCC1 via miR-363. J. Cell Mol. Med..

[48] Prager B.C., Xie Q., Bao S., Rich J.N. (2019). Cancer stem cells: The architects of the tumor ecosystem. Cell Stem Cell.

[49] Ma S., Chan K.W., Lee T.K., Tang K.H., Wo J.Y., Zheng B.J., Guan X.Y. (2008). Aldehyde dehydrogenase discriminates the CD133 liver cancer stem cell populations. Mol. Cancer Res..

[50] Wu J., Zhu P., Lu T., Du Y., Wang Y., He L., Ye B., Liu B., Yang L., Wang J. (2019). The long non-coding RNA LncHDAC2 drives the self-renewal of liver cancer stem cells via activation of Hedgehog signaling. J. Hepatol..

[51] Rinkenbaugh A.L., Baldwin A.S. (2016). The NF-kappaB pathway and cancer stem cells. Cells.

[52] Galoczova M., Coates P., Vojtesek B. (2018). STAT3, stem cells, cancer stem cells and p63. Cell Mol. Biol. Lett..

[53] Wang X., Sun W., Shen W., Xia M., Chen C., Xiang D., Ning B., Cui X., Li H., Li X. (2016). Long non-coding RNA DILC regulates liver cancer stem cells via IL-6/STAT3 axis. J. Hepatol..

[54] Grivennikov S.I., Greten F.R., Karin M. (2010). Immunity, inflammation, and cancer. Cell.

[55] Ding J., Zhao J., Huan L., Liu Y., Qiao Y., Wang Z., Chen Z., Huang S., Zhao Y., He X. (2020). Inflammation-Induced long intergenic noncoding RNA (LINC00665) increases malignancy through activating the double-stranded RNA-activated protein kinase/nuclear factor Kappa B pathway in hepatocellular carcinoma. Hepatology.

[56] Ye Y., Xu Y., Lai Y., He W., Li Y., Wang R., Luo X., Chen R., Chen T. (2018). Long non-coding RNA cox-2 prevents immune evasion and metastasis of hepatocellular carcinoma by altering M1/M2 macrophage polarization. J. Cell Biochem..

[57] Gamaev L., Mizrahi L., Friehmann T., Rosenberg N., Pappo O., Olam D., Zeira E., Bahar Halpern K., Caruso S., Zucman-Rossi J. (2021). The pro-oncogenic effect of the lncRNA H19 in the development of chronic inflammation-mediated hepatocellular carcinoma. Oncogene.

[58] Gonzalez H., Hagerling C., Werb Z. (2018). Roles of the immune system in cancer: From tumor initiation to metastatic progression. Genes Dev..

[59] Jiang R., Tang J., Chen Y., Deng L., Ji J., Xie Y., Wang K., Jia W., Chu W.M., Sun B. (2017). The long noncoding RNA lnc-EGFR stimulates T-regulatory cells differentiation thus promoting hepatocellular carcinoma immune evasion. Nat. Commun..

[60] Ji J., Yin Y., Ju H., Xu X., Liu W., Fu Q., Hu J., Zhang X., Sun B. (2018). Long non-coding RNA Lnc-Tim3 exacerbates CD8 T cell exhaustion via binding to Tim-3 and inducing nuclear translocation of Bat3 in HCC. Cell Death Dis..

[61] Yu Z., Zhao H., Feng X., Li H., Qiu C., Yi X., Tang H., Zhang J. (2019). Long non-coding RNA FENDRR acts as a miR-423-5p sponge to suppress the treg-mediated immune escape of hepatocellular carcinoma cells. Mol. Ther. Nucleic Acids.

[62] Au S.L., Wong C.C., Lee J.M., Fan D.N., Tsang F.H., Ng I.O., Wong C.M. (2012). Enhancer of zeste homolog 2 epigenetically silences multiple tumor suppressor microRNAs to promote liver cancer metastasis. Hepatology.

[63] Wei L., Chiu D.K., Tsang F.H., Law C.T., Cheng C.L., Au S.L., Lee J.M., Wong C.C., Ng I.O., Wong C.M. (2017). Histone methyltransferase G9a promotes liver cancer development by epigenetic silencing of tumor suppressor gene RARRES3. J. Hepatol..

[64] Wong C.M., Wei L., Law C.T., Ho D.W., Tsang F.H., Au S.L., Sze K.M., Lee J.M., Wong C.C., Ng I.O. (2016). Up-regulation of histone methyltransferase SETDB1 by multiple mechanisms in hepatocellular carcinoma promotes cancer metastasis. Hepatology.

[65] Long Y., Hwang T., Gooding A.R., Goodrich K.J., Rinn J.L., Cech T.R. (2020). RNA is essential for PRC2 chromatin occupancy and function in human pluripotent stem cells. Nat. Genet..

[66] Huang M.D., Chen W.M., Qi F.Z., Sun M., Xu T.P., Ma P., Shu Y.Q. (2015). Long non-coding RNA TUG1 is up-regulated in hepatocellular carcinoma and promotes cell growth and apoptosis by epigenetically silencing of KLF2. Mol. Cancer.

[67] Quagliata L., Matter M.S., Piscuoglio S., Arabi L., Ruiz C., Procino A., Kovac M., Moretti F., Makowska Z., Boldanova T. (2014). Long noncoding RNA HOTTIP/HOXA13 expression is associated with disease progression and predicts outcome in hepatocellular carcinoma patients. Hepatology.

[68] Yi T., Wang T., Shi Y., Peng X., Tang S., Zhong L., Chen Y., Li Y., He K., Wang M. (2020). Long noncoding RNA 91H overexpression contributes to the growth and metastasis of HCC by epigenetically positively regulating IGF2 expression. Liver Int..

[69] Huang G., Jiang H., Lin Y., Wu Y., Cai W., Shi B., Luo Y., Jian Z., Zhou X. (2018). lncAKHE enhances cell growth and migration in hepatocellular carcinoma via activation of NOTCH2 signaling. Cell Death Dis..

[70] Cheng J., Wei D., Ji Y., Chen L., Yang L., Li G., Wu L., Hou T., Xie L., Ding G. (2018). Integrative analysis of DNA methylation and gene expression reveals hepatocellular carcinoma-specific diagnostic biomarkers. Genome Med..

[71] Mudbhary R., Hoshida Y., Chernyavskaya Y., Jacob V., Villanueva A., Fiel M.I., Chen X., Kojima K., Thung S., Bronson R.T. (2014). UHRF1 overexpression drives DNA hypomethylation and hepatocellular carcinoma. Cancer Cell.

[72] Zhang L., Niu H., Ma J., Yuan B.Y., Chen Y.H., Zhuang Y., Chen G.W., Zeng Z.C., Xiang Z.L. (2019). The molecular mechanism of LncRNA34a-mediated regulation of bone metastasis in hepatocellular carcinoma. Mol. Cancer.

[73] Hu B., Lin J.Z., Yang X.B., Sang X.T. (2020). The roles of mutated SWI/SNF complexes in the initiation and development of hepatocellular carcinoma and its regulatory effect on the immune system: A review. Cell Prolif..

[74] Grossi E., Raimondi I., Goni E., Gonzalez J., Marchese F.P., Chapaprieta V., Martin-Subero J.I., Guo S., Huarte M. (2020). A lncRNA-SWI/SNF complex crosstalk controls transcriptional activation at specific promoter regions. Nat. Commun..

[75] Wang Y., He L., Du Y., Zhu P., Huang G., Luo J., Yan X., Ye B., Li C., Xia P. (2015). The long noncoding RNA lncTCF7 promotes self-renewal of human liver cancer stem cells through activation of Wnt signaling. Cell Stem Cell.

[76] Zhu P., Wang Y., Wu J., Huang G., Liu B., Ye B., Du Y., Gao G., Tian Y., He L. (2016). LncBRM initiates YAP1 signalling activation to drive self-renewal of liver cancer stem cells. Nat. Commun..

[77] Wang Y., Zhu P., Luo J., Wang J., Liu Z., Wu W., Du Y., Ye B., Wang D., He L. (2019). LncRNA HAND2-AS1 promotes liver cancer stem cell self-renewal via BMP signaling. EMBO J..

[78] Ribeiro D.M., Zanzoni A., Cipriano A., Ponti R.D., Spinelli L., Ballarino M., Bozzoni I., Tartaglia G.G., Brun C. (2018). Protein complex scaffolding predicted as a prevalent function of long non-coding RNAs. Nucleic Acids Res..

[79] Zhang J., Hu K., Yang Y.Q., Wang Y., Zheng Y.F., Jin Y., Li P., Cheng L. (2020). LIN28B-AS1-IGF2BP1 binding promotes hepatocellular carcinoma cell progression. Cell Death Dis..

[80] Yan X., Zhang D., Wu W., Wu S., Qian J., Hao Y., Yan F., Zhu P., Wu J., Huang G. (2017). Mesenchymal stem cells promote hepatocarcinogenesis via lncRNA-MUF interaction with ANXA2 and miR-34a. Cancer Res..

[81] Zhu P., Wang Y., Huang G., Ye B., Liu B., Wu J., Du Y., He L., Fan Z. (2016). lnc-beta-Catm elicits EZH2-dependent beta-catenin stabilization and sustains liver CSC self-renewal. Nat. Struct. Mol. Biol..

[82] Liu F., Yuan J.H., Huang J.F., Yang F., Wang T.T., Ma J.Z., Zhang L., Zhou C.C., Wang F., Yu J. (2016). Long noncoding RNA FTX inhibits hepatocellular carcinoma proliferation and metastasis by binding MCM2 and miR-374a. Oncogene.

[83] Xu F., Li C.H., Wong C.H., Chen G.G., Lai P.B.S., Shao S., Chan S.L., Chen Y. (2019). Genome-wide screening and functional analysis identifies tumor suppressor long noncoding RNAs epigenetically silenced in hepatocellular carcinoma. Cancer Res..

[84] Boque-Sastre R., Soler M., Oliveira-Mateos C., Portela A., Moutinho C., Sayols S., Villanueva A., Esteller M., Guil S. (2015). Head-to-head antisense transcription and R-loop formation promotes transcriptional activation. Proc. Natl. Acad. Sci. USA.

[85] Li Y., Syed J., Sugiyama H. (2016). RNA-DNA triplex formation by long noncoding RNAs. Cell Chem. Biol..

[86] Postepska-Igielska A., Giwojna A., Gasri-Plotnitsky L., Schmitt N., Dold A., Ginsberg D., Grummt I. (2015). LncRNA Khps1 regulates expression of the proto-oncogene SPHK1 via triplex-mediated changes in chromatin structure. Mol. Cell.

[87] Kempfer R., Pombo A. (2020). Methods for mapping 3D chromosome architecture. Nat. Rev. Genet..

[88] Xiang J.F., Yin Q.F., Chen T., Zhang Y., Zhang X.O., Wu Z., Zhang S., Wang H.B., Ge J., Lu X. (2014). Human colorectal cancer-specific CCAT1-L lncRNA regulates long-range chromatin interactions at the MYC locus. Cell Res..

[89] Rashid F., Shah A., Shan G. (2016). Long non-coding RNAs in the cytoplasm. Genom. Proteom. Bioinform..

[90] Yuan S.X., Wang J., Yang F., Tao Q.F., Zhang J., Wang L.L., Yang Y., Liu H., Wang Z.G., Xu Q.G. (2016). Long noncoding RNA DANCR increases stemness features of hepatocellular carcinoma by derepression of CTNNB1. Hepatology.

[91] Guo W., Liu S., Cheng Y., Lu L., Shi J., Xu G., Li N., Cheng K., Wu M., Cheng S. (2016). ICAM-1-related noncoding RNA in cancer stem cells maintains ICAM-1 expression in hepatocellular carcinoma. Clin. Cancer Res..

[92] Deng L., Yang S.B., Xu F.F., Zhang J.H. (2015). Long noncoding RNA CCAT1 promotes hepatocellular carcinoma progression by functioning as let-7 sponge. J. Exp. Clin. Cancer Res..

[93] Lin Y., Jian Z., Jin H., Wei X., Zou X., Guan R., Huang J. (2020). Long non-coding RNA DLGAP1-AS1 facilitates tumorigenesis and epithelial-mesenchymal transition in hepatocellular carcinoma via the feedback loop of miR-26a/b-5p/IL-6/JAK2/STAT3 and Wnt/beta-catenin pathway. Cell Death Dis..

[94] Cao C., Zhang T., Zhang D., Xie L., Zou X., Lei L., Wu D., Liu L. (2017). The long non-coding RNA, SNHG6-003, functions as a competing endogenous RNA to promote the progression of hepatocellular carcinoma. Oncogene.

[95] Huang Y., Xiang B., Liu Y., Wang Y., Kan H. (2018). LncRNA CDKN2B-AS1 promotes tumor growth and metastasis of human hepatocellular carcinoma by targeting let-7c-5p/NAP1L1 axis. Cancer Lett..

[96] Cao C., Sun J., Zhang D., Guo X., Xie L., Li X., Wu D., Liu L. (2015). The long intergenic noncoding RNA UFC1, a target of MicroRNA 34a, interacts with the mRNA stabilizing protein HuR to increase levels of beta-catenin in HCC cells. Gastroenterology.

[97] Wang H., Liang L., Dong Q., Huan L., He J., Li B., Yang C., Jin H., Wei L., Yu C. (2018). Long noncoding RNA miR503HG, a prognostic indicator, inhibits tumor metastasis by regulating the HNRNPA2B1/NF-kappaB pathway in hepatocellular carcinoma. Theranostics.

[98] Wang F., Yuan J.H., Wang S.B., Yang F., Yuan S.X., Ye C., Yang N., Zhou W.P., Li W.L., Li W. (2014). Oncofetal long noncoding RNA PVT1 promotes proliferation and stem cell-like property of hepatocellular carcinoma cells by stabilizing NOP2. Hepatology.

[99] Li Z., Zhang J., Liu X., Li S., Wang Q., Di C., Hu Z., Yu T., Ding J., Li J. (2018). The LINC01138 drives malignancies via activating arginine methyltransferase 5 in hepatocellular carcinoma. Nat. Commun..

[100] Liu J., Xu R., Mai S.J., Ma Y.S., Zhang M.Y., Cao P.S., Weng N.Q., Wang R.Q., Cao D., Wei W. (2020). LncRNA CSMD1-1 promotes the progression of hepatocellular carcinoma by activating MYC signaling. Theranostics.

[101] Jiang X., Wang G., Liu Y., Mei C., Yao Y., Wu X., Chen X., Ma W., Li K., Zhang Z. (2021). A novel long non-coding RNA RP11-286H15.1 represses hepatocellular carcinoma progression by promoting ubiquitination of PABPC4. Cancer Lett..

[102] Qin G., Tu X., Li H., Cao P., Chen X., Song J., Han H., Li Y., Guo B., Yang L. (2020). Long noncoding RNA p53-stabilizing and activating RNA promotes p53 signaling by inhibiting heterogeneous nuclear ribonucleoprotein K deSUMOylation and suppresses hepatocellular carcinoma. Hepatology.

[103] Klingenberg M., Gross M., Goyal A., Polycarpou-Schwarz M., Miersch T., Ernst A.S., Leupold J., Patil N., Warnken U., Allgayer H. (2018). The long noncoding RNA cancer susceptibility 9 and RNA binding protein heterogeneous nuclear ribonucleoprotein L form a complex and coregulate genes linked to AKT signaling. Hepatology.

[104] Ding C.H., Yin C., Chen S.J., Wen L.Z., Ding K., Lei S.J., Liu J.P., Wang J., Chen K.X., Jiang H.L. (2018). The HNF1alpha-regulated lncRNA HNF1A-AS1 reverses the malignancy of hepatocellular carcinoma by enhancing the phosphatase activity of SHP-1. Mol. Cancer.

[105] Xu J., Lu Y., Liu Q., Xia A., Zhao J., Xu X., Sun Q., Qi F., Sun B. (2020). Long noncoding RNA GMAN promotes hepatocellular carcinoma progression by interacting with eIF4B. Cancer Lett..

[106] Ryder S.D. (2003). Guidelines for the diagnosis and treatment of hepatocellular carcinoma (HCC) in adults. Gut.

[107] Tzartzeva K., Obi J., Rich N.E., Parikh N.D., Marrero J.A., Yopp A., Waljee A.K., Singal A.G. (2018). Surveillance Imaging and alpha fetoprotein for early detection of hepatocellular carcinoma in patients with cirrhosis: A meta-analysis. Gastroenterology.

[108] Lee Y.R., Kim G., Tak W.Y., Jang S.Y., Kweon Y.O., Park J.G., Lee H.W., Han Y.S., Chun J.M., Park S.Y. (2019). Circulating exosomal noncoding RNAs as prognostic biomarkers in human hepatocellular carcinoma. Int. J. Cancer.

[109] Panzitt K., Tschernatsch M.M., Guelly C., Moustafa T., Stradner M., Strohmaier H.M., Buck C.R., Denk H., Schroeder R., Trauner M. (2007). Characterization of HULC, a novel gene with striking up-regulation in hepatocellular carcinoma, as noncoding RNA. Gastroenterology.

[110] Konishi H., Ichikawa D., Yamamoto Y., Arita T., Shoda K., Hiramoto H., Hamada J., Itoh H., Fujita Y., Komatsu S. (2016). Plasma level of metastasis-associated lung adenocarcinoma transcript 1 is associated with liver damage and predicts development of hepatocellular carcinoma. Cancer Sci..

[111] Xie Z., Zhou F., Yang Y., Li L., Lei Y., Lin X., Li H., Pan X., Chen J., Wang G. (2018). Lnc-PCDH9-13:1 is a hypersensitive and specific biomarker for early hepatocellular carcinoma. EBioMedicine.

[112] Lin J., Li J., Huang B., Liu J., Chen X., Chen X.M., Xu Y.M., Huang L.F., Wang X.Z. (2015). Exosomes: Novel biomarkers for clinical diagnosis. Sci. World J..

[113] Li Y., Zhao J., Yu S., Wang Z., He X., Su Y., Guo T., Sheng H., Chen J., Zheng Q. (2019). Extracellular vesicles long RNA sequencing reveals abundant mRNA, circRNA, and lncRNA in human blood as potential biomarkers for cancer diagnosis. Clin. Chem..

[114] Kim S.S., Baek G.O., Ahn H.R., Sung S., Seo C.W., Cho H.J., Nam S.W., Cheong J.Y., Eun J.W. (2020). Serum small extracellular vesicle-derived LINC00853 as a novel diagnostic marker for early hepatocellular carcinoma. Mol. Oncol..

[115] Yuan W., Sun Y., Liu L., Zhou B., Wang S., Gu D. (2017). Circulating LncRNAs serve as diagnostic markers for hepatocellular carcinoma. Cell Physiol. Biochem..

[116] Takahashi K., Yan I.K., Wood J., Haga H., Patel T. (2014). Involvement of extracellular vesicle long noncoding RNA (linc-VLDLR) in tumor cell responses to chemotherapy. Mol. Cancer Res..

[117] Takahashi K., Yan I.K., Kogure T., Haga H., Patel T. (2014). Extracellular vesicle-mediated transfer of long non-coding RNA ROR modulates chemosensitivity in human hepatocellular cancer. FEBS Open Bio.

[118] Fu X., Zhu X., Qin F., Zhang Y., Lin J., Ding Y., Yang Z., Shang Y., Wang L., Zhang Q. (2018). Linc00210 drives Wnt/beta-catenin signaling activation and liver tumor progression through CTNNBIP1-dependent manner. Mol. Cancer.

[119] Ali M.M., Akhade V.S., Kosalai S.T., Subhash S., Statello L., Meryet-Figuiere M., Abrahamsson J., Mondal T., Kanduri C. (2018). PAN-cancer analysis of S-phase enriched lncRNAs identifies oncogenic drivers and biomarkers. Nat. Commun..

[120] Davidovich C., Cech T.R. (2015). The recruitment of chromatin modifiers by long noncoding RNAs: Lessons from PRC2. RNA.

[121] Shah M.Y., Ferrajoli A., Sood A.K., Lopez-Berestein G., Calin G.A. (2016). MicroRNA therapeutics in cancer—An emerging concept. EBioMedicine.

[122] Malakar P., Shilo A., Mogilevsky A., Stein I., Pikarsky E., Nevo Y., Benyamini H., Elgavish S., Zong X., Prasanth K.V. (2017). Long noncoding RNA MALAT1 promotes hepatocellular carcinoma development by SRSF1 upregulation and mTOR activation. Cancer Res..

[123] Ma P., Wang H., Sun J., Liu H., Zheng C., Zhou X., Lu Z. (2018). LINC00152 promotes cell cycle progression in hepatocellular carcinoma via miR-193a/b-3p/CCND1 axis. Cell Cycle.

[124] Hu W.Y., Wei H.Y., Li K.M., Wang R.B., Xu X.Q., Feng R. (2020). LINC00511 as a ceRNA promotes cell malignant behaviors and correlates with prognosis of hepatocellular carcinoma patients by modulating miR-195/EYA1 axis. Biomed. Pharmacother..

[125] You L.N., Tai Q.W., Xu L., Hao Y., Guo W.J., Zhang Q., Tong Q., Zhang H., Huang W.K. (2021). Exosomal LINC00161 promotes angiogenesis and metastasis via regulating miR-590-3p/ROCK axis in hepatocellular carcinoma. Cancer Gene Ther..

[126] Luo P., Liang C., Zhang X., Liu X., Wang Y., Wu M., Feng X., Tu J. (2018). Identification of long non-coding RNA ZFAS1 as a novel biomarker for diagnosis of HCC. Biosci. Rep..

[127] Tan J.C., Bouriakov V.D., Feng L., Richmond T.A., Burgess D. (2016). Targeted LncRNA Sequencing with the SeqCap RNA enrichment system. Methods Mol. Biol..

[128] Clark M.B., Mercer T.R., Bussotti G., Leonardi T., Haynes K.R., Crawford J., Brunck M.E., Cao K.A., Thomas G.P., Chen W.Y. (2015). Quantitative gene profiling of long noncoding RNAs with targeted RNA sequencing. Nat. Methods.

[129] Sztuba-Solinska J., Chavez-Calvillo G., Cline S.E. (2019). Unveiling the druggable RNA targets and small molecule therapeutics. Bioorg. Med. Chem..

[130] Mukherjee H., Blain J.C., Vandivier L.E., Chin D.N., Friedman J.E., Liu F., Maillet A., Fang C., Kaplan J.B., Li J. (2020). PEARL-seq: A photoaffinity platform for the analysis of small molecule-RNA interactions. ACS Chem. Biol..

[131] Arun G., Diermeier S.D., Spector D.L. (2018). Therapeutic targeting of long non-coding RNAs in cancer. Trends Mol. Med..

[132] Sun Z., Xue S., Zhang M., Xu H., Hu X., Chen S., Liu Y., Guo M., Cui H. (2020). Aberrant NSUN2-mediated m(5)C modification of H19 lncRNA is associated with poor differentiation of hepatocellular carcinoma. Oncogene.

[133] Chen M., Wong C.M. (2020). The emerging roles of N6-methyladenosine (m6A) deregulation in liver carcinogenesis. Mol. Cancer.

[134] Brown J.A., Kinzig C.G., DeGregorio S.J., Steitz J.A. (2016). Methyltransferase-like protein 16 binds the 3′-terminal triple helix of MALAT1 long noncoding RNA. Proc. Natl. Acad. Sci. USA.

[135] Wu Y., Yang X., Chen Z., Tian L., Jiang G., Chen F., Li J., An P., Lu L., Luo N. (2019). m(6)A-induced lncRNA RP11 triggers the dissemination of colorectal cancer cells via upregulation of Zeb1. Mol. Cancer.

[136] Zuo X., Chen Z., Gao W., Zhang Y., Wang J., Wang J., Cao M., Cai J., Wu J., Wang X. (2020). M6A-mediated upregulation of LINC00958 increases lipogenesis and acts as a nanotherapeutic target in hepatocellular carcinoma. J. Hematol. Oncol..

[137] Sun M., Kraus W.L. (2015). From discovery to function: The expanding roles of long noncoding RNAs in physiology and disease. Endocr. Rev..

[138] Wei L., Lee D., Law C.T., Zhang M.S., Shen J., Chin D.W., Zhang A., Tsang F.H., Wong C.L., Ng I.O. (2019). Genome-wide CRISPR/Cas9 library screening identified PHGDH as a critical driver for Sorafenib resistance in HCC. Nat. Commun..

[139] Bester A.C., Lee J.D., Chavez A., Lee Y.R., Nachmani D., Vora S., Victor J., Sauvageau M., Monteleone E., Rinn J.L. (2018). An integrated genome-wide CRISPRa approach to functionalize lncRNAs in drug resistance. Cell.

[140] Liu S.J., Horlbeck M.A., Weissman J.S., Lim D.A. (2021). Genome-scale perturbation of long noncoding RNA expression using CRISPR interference. Methods Mol. Biol..

